# Hexagonal tungsten oxide nanoflowers as enzymatic mimetics and electrocatalysts

**DOI:** 10.1038/srep40928

**Published:** 2017-01-27

**Authors:** Chan Yeong Park, Ji Min Seo, Hongil Jo, Juhyun Park, Kang Min Ok, Tae Jung Park

**Affiliations:** 1 Department of Chemistry, Chung-Ang University, 84 Heukseok-ro, Dongjak-gu, Seoul 06974, Republic of Korea; 2 School of Chemical Engineering and Materials Science, Chung–Ang University, 84 Heukseok–ro, Dongjak–gu, Seoul 06974, Republic of Korea

## Abstract

Tungsten oxide (WO_*x*_) has been widely studied for versatile applications based on its photocatalytic, intrinsic catalytic, and electrocatalytic properties. Among the several nanostructures, we focused on the flower-like structures to increase the catalytic efficiency on the interface with both increased substrate interaction capacities due to their large surface area and efficient electron transportation. Therefore, improved WO_*x*_ nanoflowers (WONFs) with large surface areas were developed through a simple hydrothermal method using sodium tungstate and hydrogen chloride solution at low temperature, without any additional surfactant, capping agent, or reducing agent. Structural determination and electrochemical analyses revealed that the WONFs have hexagonal Na_0.17_WO_3.085_·0.17H_2_O structure and exhibit peroxidase-like activity, turning from colorless to blue by catalyzing the oxidation of a peroxidase substrate, such as 3,3′,5,5′-tetramethylbenzidine, in the presence of H_2_O_2_. Additionally, a WONF-modified glassy carbon electrode was adopted to monitor the electrocatalytic reduction of H_2_O_2_. To verify the catalytic efficiency enhancement by the unique shape and structure of the WONFs, they were compared with calcinated WONFs, cesium WO_*x*_ nanoparticles, and other peroxidase-like nanomaterials. The results indicated that the WONFs showed a low Michaelis-Menten constant (*k*_*m*_), high maximal reaction velocity (*v*_*max*_), and large surface area.

Functional systems are roughly classified into (i) man-made (artificial) and (ii) naturally occurring (mainly biologically related) systems^1^. In the case of the former, metal oxides have been widely introduced for the use of the structural, chemical, and physical properties. As the transition metal oxides are generally dependent on their synthesis conditions, the knowledge of the relationships between the synthesis conditions for a specific metal oxide and its functional properties is important to obtain the optimal properties for a given application^2^,^3^,^4^,^5^,^6^,^7^. Since Fe_3_O_4_ magnetic nanoparticles (MNPs) were first found to possess unexpected enzyme-like activity^8^, zero-dimensional nanomaterials such as cerium oxide^9^, gold nanoparticles^10^, carbon nanodots^11^, and ZnFe_2_O_4_ MNPs^12^, one-dimensional nanomaterials such as V_2_O_5_ nanowires^13^ and two-dimensional nanomaterials such as graphene oxide^14^, carbon nitrides^15^, and inorganic–organic hybrid materials^16^ have been exploited as peroxidase mimics for catalyzing H_2_O_2_-mediated color change reactions or as electrocatalysts. These nanomaterials have emerged as a new class of ideal catalyst and have been applied as powerful tools for bioassays and medical diagnostics due to their low cost, high stability, easy preparation, controllable structure and composition, and tunable catalytic activity^17^,^18^.

Among the various nanomaterials, tungsten oxide (WO_*x*_), an n-type indirect band gap semiconductor, has attracted much interest because of its outstanding physicochemical properties and photo- and electro-catalytic properties. Due to their unique properties, WO_*x*_ has been applied in electrochromic or photochromic devices, secondary batteries, gas sensors and as a catalyst and electrocatalyst. Several previous studies related to WO_*x*_ application have been found over the last decade^19^,^20^,^21^,^22^,^23^. The morphology of WO_*x*_ is highly dependent on the pH of the reaction system. According to previous work^24^, WO_*x*_ adopted to a rod shape at pH 3.0, a wheel-like shape at pH 2.0, and a flower-like shape at pH 1.5, respectively. Since the catalytic activity is closely related to both the surface area and surface chemistry of a catalyst, we have focused on the flower-like shape synthesis. Because the random branches of the nanoflowers provide both a large surface area and greater substrate interaction without sacrificing good electron transportation^25^,^26^. There are three keys to obtain the improved flower-shaped WO_*x*_ (WONFs) with a large surface area. First is acidic environment (pH 1.6), seconds is the synthesis was conducted at a low temperature (to provide a low reaction rate), and last is high precursor concentration. Like hexagonal zinc oxide nanorods, hexagonal WO_*x*_ crystals have been considered polar crystals with ±(0001) polar planes. In particular, these polar crystals are prone to growing along their polar directions (c-axis) at a low growth rate. The difference in crystal growth rate between the polar plane and nonpolar plane would produce anisotropic crystal growth^27^,^28^.

Another factor influencing the crystal morphology is the capping agent, which can selectively adsorb onto the preferred crystal planes and regulate the crystal growth rate. In this reaction system, NaCl can act as a capping reagent by adsorbing onto the crystal plane parallel to the c-axis of the WO_3_ crystal nucleus. For this reason, previous studies^24^,^29^ have added NaCl to obtain rod- or flower-shaped WO_*x*_. However, in our study (Fig. 1), the use of a higher concentration of the sodium tungstate precursor obviates the need to add extra NaCl. Moreover, Cs_2_WO_4_-based experiments were performed to investigate the influence of the Na^+^ cation on the overall structure of WO_*x*_; however, the resulting nanoparticles, Cs_0.3_(WO_3_), were not flower-shaped and denoted as CsWONPs. The WONFs were compared with CsWONPs in terms of structural and catalytic properties.

The as-synthesized WONFs were characterized by X-ray diffraction (XRD), thermogravimetric analysis (TGA), differential scanning calorimetry (DSC), scanning electron microscopy (SEM), transmission electron microscopy (TEM), energy dispersive X-ray spectroscopy (EDAX), Brunauer-Emmett-Teller (BET) analysis, Fourier transform infrared (FT-IR) spectroscopy, and X-ray photoelectron spectroscopy (XPS). To verify the influence of heat on their structure, WONFs were calcinated over their crystallization temperature (600 °C) as determined by DSC. This sample, denoted as cWONFs, was compared with WONFs. Additionally, it was manifested that the WONFs exhibit peroxidase-like activity and can be used as a H_2_O_2_ sensor in both colorimetric and electrochemical techniques. For optical detection, the 3,3′,5,5′-tetra-methylbenzidine (TMB)-assisted color change method was adopted to a glassy carbon electrode (GCE) modified with WONFs and then coated with Nafion (Nf) to enhance the ionic conductivity in electrochemical analyses, such as cyclic voltammetry (CV) and chronoamperometry^30^,^31^.

## Results and Discussion

### Structural properties of WONFs

**Figure 2:**
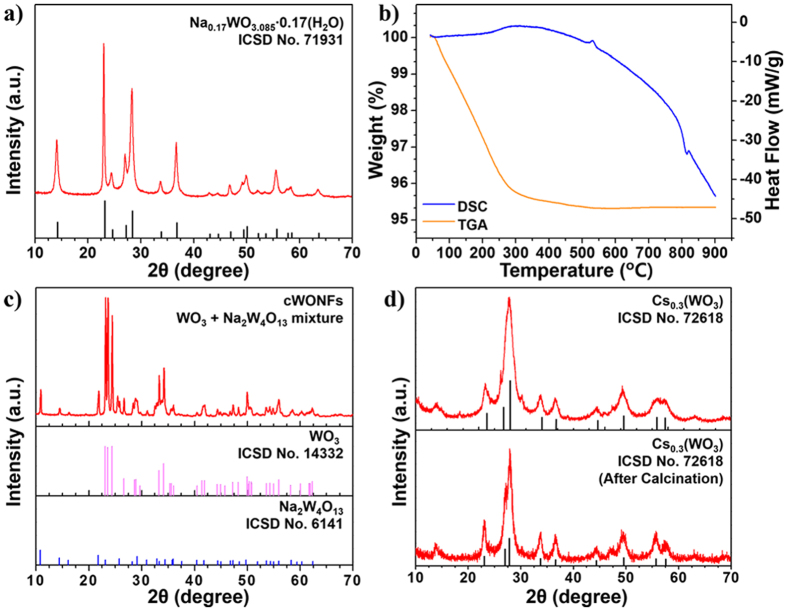
(**a**) Powder XRD pattern, (**b**) TGA (orange) and DSC (blue) curves for WONFs. Powder XRD patterns of (**c**) cWONFs and (**d**) CsWONPs and cCsWONPs.

Figure 2a shows the XRD patterns of WONFs, which are well matched by the pattern for hexagonal Na_0.17_WO_3.085_ ∙ 0.17H_2_O (h-WO_3_, ICSD No. 71931, hexagonal, *P*6/*mmm*, a = 7.33 Å, c = 3.89 Å)^32^. There are no peaks for any other phase or impurity. Because the structure of h-WO_3_ has been reported elsewhere^33^, the only a very brief structural description will be given. WONFs have been classified within the hexagonal tungsten bronze (HTB) family. As shown in Fig. S1a, the structural model of HTB can be generalized as A_*x*_WO_3_ (A = an alkali metal and *x* ≈ 0.3). The three-dimensional framework is composed of corner-shared WO_6_ octahedral that maintain a hexagonal backbone. Within this framework, three- (3-MR) and six-membered ring (6-MR) channels exist.

Both Na^+^ cations and water molecules reside in the 6-MR channels of WONFs. To understand the relevant physical properties of the WONFs, thermal analysis was performed. Figure 2b shows the TGA and DSC curves of the sample. From the TGA curve, weight loss began at room temperature and reached completion at 400 °C, and no further weight loss or gain is observed up to 900 °C. Of the total weight loss (4.5 wt%), 1.27 wt% is attributed to the theoretical amount of H_2_O molecules intercalated in the h-WO_3_ crystal structure, and the rest is attributed to the release of adsorbed and chemisorbed water molecules on the surface of WONFs^34^. These results can be verified by broad DSC curve in range of 50 to 500 °C. The total weight loss herein is greater than the reported value due to the large surface area of WONFs^35^. Based on the DSC results, the crystallization temperature was confirmed as 525 °C. To reveal the morphology and structural changes of WONFs at that temperature, the sample was calcinated at 600 °C for 6 h in an alumina crucible. Figure 2c is an XRD pattern of the cWONFs. As the WONFs were calcinated, no water molecules were intercalated in the crystals; the resulting cavities caused the other atoms, namely, W, O, and Na, to bind with each other. As a result, the composition of the WONFs changed markedly, having a high proportion of WO_3_ and a relatively small proportion of Na_2_W_4_O_13_, as the crystal structure of WONFs (Na_0.17_WO_3.085_ ∙ 0.17H_2_O) contains few Na^+^ cations, the source of Na_2_W_4_O_13_^35^. The structural models of Na_2_W_4_O_13_ and WO_3_ are presented in Fig. S1b and S1c, respectively. Meanwhile, Na_2_W_4_O_13_ adopted a layered structure, and WO_3_ adopted a monoclinic structure. Figure 2d shows the XRD patterns of hexagonal CsWONPs (Cs_0.3_WO_3_) and calcinated CsWONPs (cCsWONPs). These patterns are well-matched by the pattern of Cs_0.3_(WO_3_) (ICSD No. 72618, hexagonal, *P*6_3_/*mcm*, a = 7.41 Å, c = 7.61 Å), lacking impurities or other phases^36^. Unlike WONFs, CsWONPs did not form a hydrate. Because the ionic radius of Cs is larger than that of Na, there is no space for H_2_O molecules in the hexagonal channel. Therefore, the d-spacing of CsWONPs is slightly larger than that of WONFs. The TGA and DSC curves for CsWONPs reveals weight loss began at room temperature and reached completion at 300 °C for this sample (Fig. S2) and, being maintained thereafter. Because no specific crystallization temperature was observed, the sample was calcinated in an alumina crucible under the same conditions as WONFs (600 °C, 6 h). The weight loss was 3 wt%, attributed to the release of adsorbed water molecules due to the similarity of the XRD patterns of CsWONPs and cCsWONPs. Figure S1d represents the structural model of the CsWONPs.

### Morphological studies of WONFs

**Figure 3:**
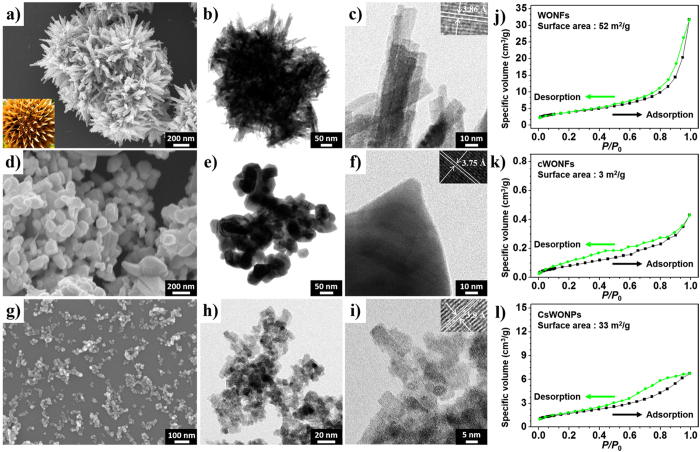
FE-SEM images of (**a**) WONFs, (**d**) cWONFs, and (**g**) CsWONPs. TEM images of (**b,c**) WONFs, (**e,f**) cWONFs, and (**h,i**) CsWONPs. Insets of picture represent the detailed d-spacing of each crystal structure. Nitrogen adsorption isotherm results for (**j**) WONFs, (**k**) cWONFs, and (**l**) CsWONPs.

Figure 3a,d and g present SEM images of synthesized WONFs, cWONFs, and CsWONPs, respectively. While WONFs have a flower-like shape, cWONFs lack this morphology, because the nanorods comprising the flower petals collapse when the crystallization temperature (525 °C) is exceeded. The rods are approximately 250–400 nm length and approximately 8–15 nm width. Meanwhile, unlike WONFs, CsWONPs consist of irregularly shaped, agglomerated particles. This anisotropic growth on the WO_3_ could result from the alkali metal ions interfering with the formation of the WO_3_ morphology. Therefore, the SEM images imply that the presence of different alkali metal ions affects the structure and surface area of the formed WO_*x*_. To monitor the influence of the initial precursor concentration to their morphology, the synthesis was conducted under different concentrations of sodium tungstate (0.1 M, 0.3 M, 0.5 M, and 1.0 M) while the other conditions such as pH, temperature, and reaction time were fixed. The SEM images of them are shown in Fig. S3. Despite their different morphologies, the samples had similar XRD patterns of h-WO_3_ (Fig. S4) except when 0.1 M of Na_2_WO_4_ ∙ 2H_2_O was introduced. These results indicate that 0.3 M of initial precursor is adequate for constructing the flower-like morphology in this regard both amounts of surfactants and tungstates^37^. However, at low concentrations, it forms the mixture of orthorhombic WO_3_(H_2_O) (ICSD No. 201806) and h-WO_3_ due to the shortage of capping agent Na^+^ cation, which is the key factor for c-axis growth in tungsten oxide^29^. In contrast, when initial precursor concentration was over 0.3 M, these crystals formed the agglomerated shape. Because hexagonal-phase WO_3_ is a metastable phase, that is similar with why the structure directing agent is strongly required to prohibit the aggregation^38^. In other words, as the portion of the tungstate anion overcomes the Na^+^ cation, the violent aggregation was induced.

Next, to investigate the influence of the reaction temperature, WO_*x*_ was synthesized under 50 °C, 60 °C, 80 °C, 100 °C, 150 °C, and 200 °C, respectively. These different synthesized WO_*x*_ nanomaterials appeared the different shapes (Fig. S5a–e), except 50 °C (nothing is synthesized at this temperature). As the reaction temperature increase, h-WO_3_ rods become shorts and agglomerated. Figure S6 shows the XRD patterns of these samples. In the cases of samples synthesized at 60 °C, 80 °C, and 100 °C are well matched with h-WO_3_. However, at the case over 150 °C, it builds the mixture of monoclinic WO_3_ (ICSD No. 31823) and orthorhombic WO_3_ ∙ 1/3(H_2_O) (ICSD No. 82941), which means these phases are more thermodynamically stable than hexagonal phase^39^. Figure S5f is a table for the surface area of differently synthesized WO_*x*_. From that, when it synthesized under low temperatures, c-axis growth is obviously dominant and more beneficial to increase the surface area.

Figure 3b,e and h are typical TEM images of the prepared WONFs, cWONFs, and CsWONPs, respectively. The high-resolution TEM images of WONFs, cWONFs, and CsWONPs show the detailed d–spacing, and the average interplanar distances for these samples, which are 3.86 ± 0.13 Å (Fig. 3c), 3.75 ± 0.11 Å (Fig. 3f), and 3.90 ± 0.09 Å (Fig. 3i), respectively. These results are individually similar with the XRD results at (001) diffraction. In particular, (001) diffraction of cWONFs is smaller than that of WONFs, suggesting that the intercalated H_2_O molecules were released from the 6-MR channel. On the other hand, the (001) diffraction of CsWONPs is slightly larger than that of WONFs because of the larger size of the intercalated alkali cations. The WONFs, cWONFs, and CsWONPs were subjected to EDAX analysis to verify the presence of Na, W, and O (Fig. S7). The copper (Cu) peaks on the spectrum (approximately 8 keV) originated from the copper grid used for the analysis.

The multi-point BET plots for the WONFs (Fig. 3j), cWONFs (Fig. 3k), and CsWONPs (Fig. 3l) represent that the above-mentioned morphological differences directly affect the surface area. At the beginning of the adsorption step, nitrogen gas molecules adsorb onto the nanocrystals, forming a monolayer. Next, additional gas molecules stack onto this layer to form a multilayer due to their high intermolecular affinities. After the formation of the monolayer via molecular absorption, the adsorption of the subsequent layers can be investigated from a specified end point of the monolayer by purging the reaction vessel used for the BET analysis with N_2_ gas at 1 atm. As a result, data from a specific stage of the adsorption process (0.05–0.35 *P/P*_*0*_) was chosen to obtain the surface area of each sample corresponding to the monolayer^40^. For the WONFs sample, the surface area was 52 m^2^/g, and a slight hysteresis was observed due to their shape. The flower morphology of this sample (Fig. 3a) included many exposed petals which provided a large surface area. In turn, this large surface area allowed N_2_ molecules to easily adsorb onto the petals. However, the gas molecules located in the core sites were less easily desorbed due to capillary effects, creating a hysteresis. Meanwhile, cWONFs have a lower surface area (3 m^2^/g) than WONFs. As the rods in WONFs collapse during their calcination into cWONFs, their actual contact area decreases. This sample also shows a slight hysteresis owing to its rough surface morphology. Finally, the CsWONPs samples have a relatively large surface area (33 m^2^/g). Unlike the two nanocrystals described above, CsWONPs present a clear hysteresis, which indicates that this nanocrystal has a mesoporous structure. Although the WONFs had the largest particle size among the materials studied, their surface area was also the largest due to their flower-like shape. Table S1 compares the surface areas of the WONFs and the other WO_*x*_-based nanocrystals.

From the Table S1, this work shows the better performance as the surface area. There are three major differences in synthetic condition between the previous work and this work. First is the amount of precursors, second is whether to add the extra NaCl, and third is reaction temperature and time. The molecular weight of Na_2_WO_4_·2H_2_O is 605.65 g/mol, and the molar concentration of the above system is approximately 18 mM. Moreover, they added some NaCl to obtain the rod-shaped crystals. However, in spite of no adding the NaCl, we attained the flower-shaped crystals. Due to the use of a higher concentration of the sodium tungstate precursor (0.3 M), additional NaCl is not needed. Lastly, we synthesized at a low temperature to promote the anisotropic growth (in z-axis) of WO_*x*_ crystals, and we extended the reaction time to compensate the low growth rate.

These strategical ideas are based on the formation mechanism of WONFs, and it can be explained by reported formation mechanism of WO_3_ nanorods^29^. Their reactions are belows:


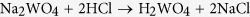



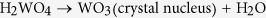



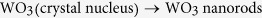


The HCl solution was added to Na_2_WO_4_ solution, H_2_WO_4_ was subsequently formed. When the synthetic temperature overcomes the decomposition temperature of H_2_WO_4_ (over 60 °C determined by this work), the nucleation process was started, and WO_3_ as a crystal nucleus is formed. Since WO_3_ nuclei bear the negative charge, Na^+^ ions easily adsorb onto their surfaces. As a result, this nuclei formation rate is very slow due to the low temperature, but it can easily create the large-sized nuclei, which is the fundamental for the flower-shaped crystal, and help to anisotropic growth. In the case of WO_3_, these species are consumed by forming the nuclei, but Na^+^ are relatively used in small amounts. Thus, simple increase as the precursor concentration can build the flower-shape.

### Chemical Investigation of WONFs

**Figure 4:**
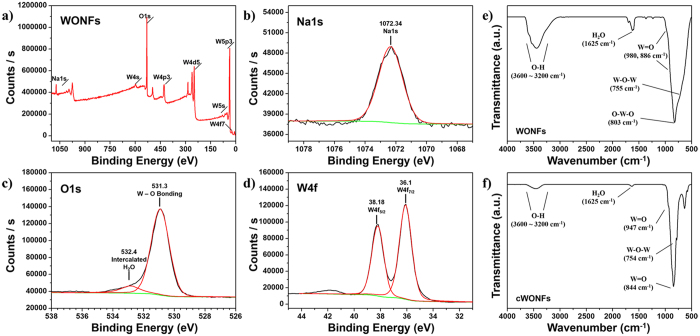
(**a–d**) XPS spectra of WONFs. FT-IR spectra of (**e**) WONFs and (**f**) cWONFs dispersed in KBr pellets.

To obtain more information on the structure and chemical composition of the synthesized materials, XPS and IR spectroscopy were utilized. The XPS spectra (Fig. 4a–d) show that the W4f peaks located at 36.1 eV and 38.18 eV can be attributed to W4f_7/2_ and W4f_5/2_, respectively, which result from the spin orbit splitting of 4f_7/2_ with 4f_5/2_. This value is in good agreement with the previously reported values^41^,^42^. These two peaks are well separated, without any shoulder, which indicates that almost all W atoms are in the +6 oxidization state. The O1s peak is located at 531.3 eV, which is ascribed to the W–O peak, and a shoulder peak at 532.4 eV is due to the oxygen in water molecules intercalated in the WONFs crystal structure^43^. The Na1s peak at 1072.34 eV is consistent with the +1 oxidation state of sodium^44^. The chemical composition of WONFs was calculated by dividing the peak intensities into the reported sensitivity factors for each element^45^. The composition ratio is (Found: Na, 0.12; W, 1; O, 3.65. Calc. for Na_0.17_WO_3.085_ ∙ 0.17H_2_O: Na, 0.17; W, 1; O, 3.255). Notably, the calculated oxygen content is higher than that predicted by the chemical formula for WONFs. Because WONFs have a large surface area, a large number of H_2_O molecules are adsorbed on their surface, affecting the XPS results^46^. In the case of cWONFs (Fig. S8), the tendency is similar with WONFs, but the oxygen portion is slightly higher than WONFs and there is not H_2_O shoulder peak. Moreover, the composition ratio of cWONFs was found to be Na, 0.16; W, 1; O, 3.73. Because cWONFs are mixtures of Na_2_W_4_O_13_ and WO_3_, their calculated ratios were 11.8% for the Na_2_W_4_O_13_ and 78.2% for the WO_3_, respectively.

The FT-IR spectra of the WONFs and cWONFs are shown in Fig. 4e and f, respectively. The band in v_max_/cm^−1^ 3600–3200 (O-H stretching) and 1625 (H_2_O bending) supports the presence of coordinated H_2_O molecules in WONFs. In addition, the fingerprint region (<1000 cm^−1^ range) includes W-O related peaks; for example, the shoulders at v_max_/cm^−1^ 980 and 886 (W = O), 817 (O-W-O stretching), and 755 (W-O-W bending)^47^. Compared with WONFs, the intensities of the H_2_O-related bands of cWONFs, such as those at 3600–3200 cm^−1^ (O-H stretching) and 1625 cm^−1^ (H_2_O bending), decreased markedly. The slightly remaining bands are due to H_2_O molecules adsorbed on their surfaces. Moreover, after calcination of the WONFs, W- and O-related bands such as W = O stretching and W-O-W bending bands are slightly shifted. Unlike WONFs, as cWONFs have not any cavity in their crystal system, bonding energies are increase and this phenomenon is subsequently induced.

### Assessment of the peroxidase-like activity of WONFs and its kinetics

**Figure 5:**
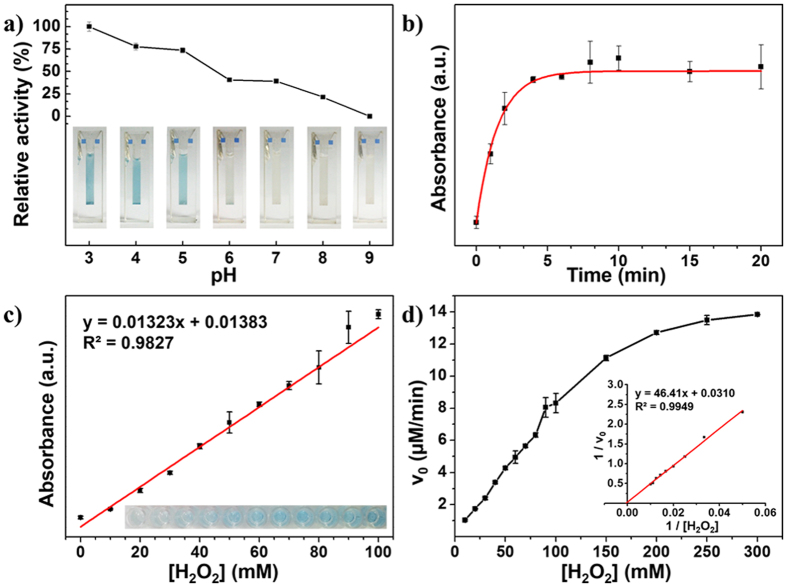
(**a**) pH-dependent absorbance changes at 450 nm. Insets is the corresponding photos for different pH values (from left to right: 3.0–9.0). (**b**) UV/vis absorbance spectra of the material resulting from TMB oxidation under WONFs as a function of time. (**c**) Dose-response curve for different concentrations of H_2_O_2_ using WONFs. The inset represents the corresponding photos for different concentrations of H_2_O_2_ (from left to right: 0–100 mM). (**d**) Steady-state kinetic analyses using the Michaelis-Menten model and Lineweaver-Burk model (inset) for WONFs with varying H_2_O_2_ concentration.

To show the peroxidase-like activity of WONFs, a colorimetric analysis was conducted under the conditions optimized as follows: Fig. 5a presents the pH-dependent responses. From the results, when using WONFs in a high-pH environment, the TMB solution does not become blue, as this transition requires this molecule to be in its oxidized form (ox-TMB). However, under basic conditions, this molecule is deprotonated, and the color change does not occur^48^. Therefore, weakly acidic conditions (pH 3.0) were chosen. Figure 5b represents the time-dependent response after adding H_2_SO_4_ solution at 0, 1, 2, 4, 6, 8, 10, 15, and 20 min. After 5 min, the steady-state is reached; therefore, the incubation time was fixed at 5 min. This result indicates that the catalytic oxidation of TMB by WONFs follows Michaelis-Menten behavior. Figure 5c shows the effect of H_2_O_2_ concentration on the bio-mimetic sensors under optimal conditions, namely, pH 3 and 5 min of incubation time. The absorbance increased as the H_2_O_2_ concentration from 0 to 100 mM. The relationship was linear (R^2^ = 0.9827), and the detection limit was 138 μM. In contrast, cWONFs and CsWONPs did not work (Fig. S9).

Based on the above results, the steady-state kinetic assays for WONFs were examined. The Michaelis-Menten kinetics of the enzymatic reactions is given by Eq. (1).


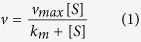



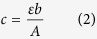



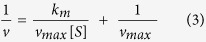


In the equation, *v* is the initial reaction rate, *k*_*m*_ is the Michaelis-Menten constant, *v*_*max*_ is the maximal reaction velocity, and [S] is the substrate concentration. The value of *k*_*m*_ is equal to the substrate concentration at half of *v*_*max*_, and enzymes with smaller *k*_*m*_ have higher affinity with the substrate^49^. To obtain the initial rate, the oxidized TMB concentration was quantified by Lambert-Beer’s law in Eq. (2) and then divided by 5 min. Here, A is the absorbance after 5 min of reaction; b is the path length of the absorbing medium (here, 0.606 cm), ε is the absorbance coefficient of the ox-TMB and *λ*_*max*_(ox-TMB)/nm 450 (mol^−1^ cm^−1^ 590,000)^50^, and c is the concentration of ox-TMB. Figure 5d is the typical Michaelis-Menten curves under optimal conditions over a range of concentrations with one substrate. To determine *v*_*max*_ and *k*_*m*_ more precisely, their double reciprocal plots, corresponding to the Lineweaver-Burk equation^51^ in Eq. (3), were adopted (inset of Fig. 5d). The calculated kinetic parameters of WONFs were compared with those of HRP and various nanomaterials with peroxidase-like activity, such as AgVO_3_, Fe_3_O_4_ MNPs, ZnFe_2_O_4_ MNPs, Co_3_O_4_ NPs, and GO-COOH (Table S2). Among them, WONFs have the smallest *k_m_* value and largest *v*_*max*_ value for H_2_O_2_.

### Electrochemical analysis of WONFs and H_2_O_2_ detection

**Figure 6:**
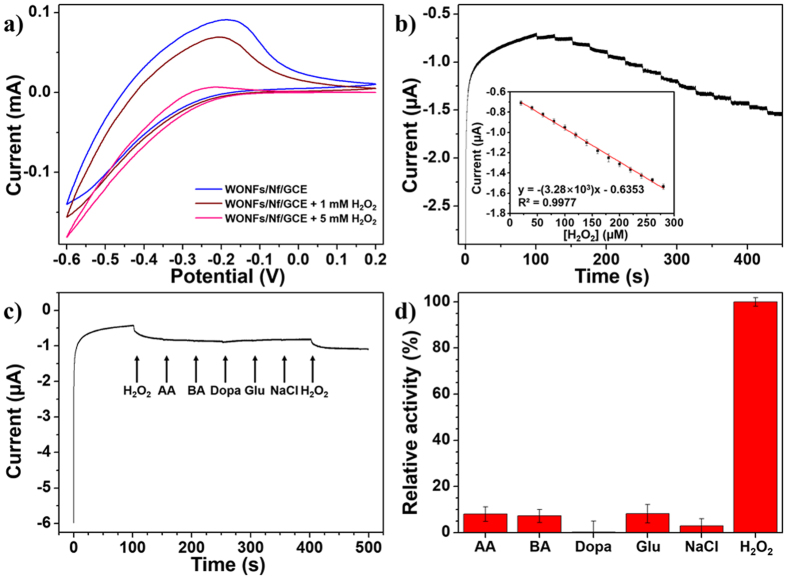
(**a**) CV spectra of different modified GCEs (Bare GCE; GCE/Nf; WONFs/Nf/GCE; WONFs/Nf/GCE+1mM H_2_O_2_; WONFs/Nf/GCE+5 mM H_2_O_2_) in acetate buffer solution (pH 3.0) at a scan rate of 20 mV/s. (**b**) Chronoamperometric current responses of the WONFs/Nf/GCE at 200 μL of 1 mM successive addition of H_2_O_2_ at −0.3 V. The inset of graph is the response current for different concentrations of H_2_O_2_. (**c**) Chronoamperometry of WONFs/Nf/GCE *vs*. Ag/AgCl after the addition of H_2_O_2_ and interferents: ascorbic acid (AA), boric acid (BA), dopamine (Dopa), Glucose (Glu), and NaCl in 20 mM acetate buffer (pH = 3.0) at an applied potential of −0.1 V. (**d**) Relative current responses of each interferents and H_2_O_2_.

Figure 6a shows the CV responses of different electrodes modified with WONFs/Nf/GCE, WONFs/Nf/GCE + 1 mM H_2_O_2_, and WONFs/Nf/GCE + 5 mM H_2_O_2_ to investigate the changes in their electrochemical behaviors. For the bare GCE (Fig. S10), regardless of Nf coating or the addition of H_2_O_2_, no redox responses were observed in the potential range. However, after modification by WONFs, a reduction peak was observed at −0.25 V, and upon the addition of H_2_O_2_ to WONFs/Nf/GCE, its reduction current decreased with increasing H_2_O_2_ concentration because of its electrocatalytic effect^52^,^53^. Figure S11 shows the CV spectra of WONFs/Nf/GCE with H_2_O_2_ at different pH values (3.0–6.0); these results represent a similar trend to the results of the colorimetric detection experiment. Among the different tested pH values, pH 3.0 was chosen due to its specific current response and good linearity (Fig. S12). In order to monitor the electrocatalytic activity of this WONFs/Nf/GCE in real-time, it was also studied by chronoamperometry at −0.25 V with successive addition of 20 μM H_2_O_2_ (Fig. 6b) every 25 s. Upon the addition of H_2_O_2_ to this system, the reduction current increased rapidly within 3 s, and the limit of detection (LOD) was 56.0 nM. This value is lower than that obtained using the colorimetric detection. Figure 6c shows the chronoamperometric curve for WONFs/Nf/GCE *vs*. Ag/AgCl with such interferents as ascorbic acid (AA), boric acid (BA), dopamine (Dopa), glucose (Glu), and NaCl to assess the selectivity of WONFs. The results reveal that WONFs have excellent selectivity for H_2_O_2_, as the responses to the interferents were less than 12% (Fig. 6d). Figure S13 is the CV spectra of cWONFs. After calcination, its reduction peaks current decreased dramatically from the mA to the μA scale due to its small surface area and different lattice composition. From the above results, it was revealed that these products also have catalytic properties. WONFs and CsWONPs present the slightly higher value than cWONFs. While CsWONPs and WONFs have hexagonal structures, cWONFs are mixtures of monoclinic and triclinic structure. Consequently, cWONFs are not working due to the different crystal system and relatively smaller surface area. In the case of CsWONPs, even though their current and surface area are almost half than WONFs, it also didn’t work for the optical detection system. In the case of WONFs, they composed with rod-shaped petals which retain the high surface energy than others due to {001} facet^54^. However, CsWONPs has a spherical shape. Thus, this differences affect to their activities via the surface area and energy level.

In summary, WONFs have been synthesized using a simple hydrothermal method without surfactants, capping agents, or reducing agents. To obtain the improved flower-like shape, the synthesis was conducted under low temperature, high precursor concentration, and low pH. The WONFs were characterized using a combination of experimental techniques, and it was found that WONFs have the largest surface area (52 m^2^/g) despite having a particle size larger than that of CsWONPs. Furthermore, WONFs have intrinsic peroxidase-like activity and electrocatalytic properties. For colorimetric detection, TMB was adopted, and the LOD was 138 μM within a linear range of 0–100 mM. When using electrochemical methods, a modified GCE was used, and the LOD was 56.0 nM within a linear range of 0–280 μM. In particular, the LOD obtained when using WONFs in electrochemical sensing is lower than that obtained when using WONFs in colorimetric detection. Additionally, the characteristics and catalytic activity of cWONFs and CsWONPs were studied. However, neither material exhibited both intrinsic peroxidase-like activity and good electrocatalytic activity due to the different crystal systems and morphologies. From the results, our strategy to obtain the flower-shaped WO_*x*_ which consisted with well-separated rods as petals was valid for H_2_O_2_-catalytic systems.

## Methods

### Chemical reagents

Sodium tungstate dihydrate (Na_2_WO_4_ ∙ 2H_2_O), 5% (w/v) Nf in perfluorinated resin solution, TMB, and dopamine hydrochloride were purchased from Sigma-Aldrich (St. Louis, MO). Cesium tungsten oxide (Cs_2_WO_4_, 99.9%) was purchased from Alfa Aesar (Ward Hill, MA). Hydrochloric acid (35.0–37.0%) and L(+)-ascorbic acid were purchased from Samchun (Seoul, Korea). Boric acid was purchased from Kanto Chemical (Tokyo, Japan). D(+)-glucose and NaCl were purchased from Junsei (Tokyo, Japan). H_2_O_2_ (30%) was purchased from Daejung (Siheung, Korea). All reagents were used as received and diluted with deionized (DI) water (Direct–Q^®^ 3 Water Purification System, 18 MΩ, Millipore, Billerica, MA). A 1% of Nf solution was prepared by dissolving 3 μl of Nf in 12 μl of isopropanol (Merck, Darmstadt, Germany).

### Preparation of Na_0.17_WO_3.085_ ∙ 0.17H_2_O nanoflowers

The WONFs were synthesized as follows: First, 0.7 M of HCl solution was carefully added to 15 ml of 0.3 M Na_2_WO_4_ ∙ 2H_2_O solution until the pH of the clear solution was 1.6. The obtained solution was transferred into a Teflon-lined 20-mL capacity autoclave and subsequently sealed. The autoclave was heated at 60 °C for 48 h and then allowed to cool at room temperature. Next, the white precipitate was isolated by centrifugation and decantation. Finally, the product was washed with DI water three times and dried for 24 h at 60 °C. Different experiments were conducted in parallel with a tunable amount of Na_2_WO_4_ ∙ 2H_2_O and at different reaction temperatures. The above described method was also used for the preparation of CsWONPs by using Cs_2_WO_4_.

### Characterizations

The powder XRD patterns of the samples were recorded using a D8-Advance instrument from Bruker AXS (Karlsruhe, Germany) with Cu Kα radiation at room temperature (40 kV and 40 mA). The 2*θ* range was 10–70° with a step size of 0.2° and step time of 0.55 s. TGA was performed under an Ar flow at a heating rate of 10 K/min from room temperature to 900 °C with a TGA N-1000 from Scinco (Seoul, Korea). DSC was performed under same conditions as TGA using a Setaram Labsys Evo thermal analyzer (Setaram, Cailure, France). The size and morphology of the WONFs were characterized by SEM and TEM. SEM images were recorded on SIGMA instrument from Carl Zeiss (Cambridge, UK). The samples were dried overnight at room temperature. As the prepared samples had low conductivity, they were coated with a Pt using a sputter-coater (COXEM, KIC-1A, Seoul, Korea) to avoid charge-up before analysis. TEM images were obtained on a Tecnai G2 F30 S–TWIN instrument from FEI (Hillsboro, OR). The samples for TEM analysis were prepared by dispersing WONFs in DI water and placing a droplet of the solution on a copper grid with a 300 nm mesh and 3 mm diameter. Elemental analysis of the WONFs was conducted using an EDAX analyzer coupled with TEM. FT-IR spectra of WONFs dispersed in KBr pellets were recorded from 4000–400 cm^−1^ on an IFS66V/S & HYPERION 3000 instrument from Bruker Optics (Billerica, MA). XPS spectra were recorded on a MultiLab 2000 instrument from Thermo Scientific (Waltham, MA). The surface areas of the WONFs were determined by BET analysis (Quantachrome, Nova 1200e, Boynton Beach, FL) from N_2_ gas adsorption data using the multi-point BET technique. The samples were dehydrated at 60 °C for 6 h before being analyzed.

### Assessment of the peroxidase-like activity of WONFs and its kinetics

To show the peroxidase-like activity of WONFs, the following procedure was used. First, the absorbance was recorded at different pH conditions (pH 3.0–9.0) to monitor the pH effect. Second, the incubation time was optimized by varying the reaction time. Third, the absorbance was recorded at different concentrations of H_2_O_2_ (0–100 mM) to examine the effect of H_2_O_2_ concentration on the peroxidase-like activity of WONFs. Finally, the kinetic studies were conducted using the above conditions. In brief, 50 μl of well-dispersed WONFs (1 mg/ml), 50 μl of TMB (5 mM), 50 μl of buffer solution, and 300 μl of DI water were mixed together. The TMB was first dissolved in DMSO and then diluted with DI water. Then, 50 μl of H_2_O_2_ (10 mM) was added to the solution. In order to find the optimal pH, acetate buffer (20 mM) was used in range of the pH 3.0–5.0, and Tris-Cl buffer (20 mM) was used in range of the pH 6.0–9.0. The mixtures were incubated at 60 °C for 5 min. At last, 50 μl of H_2_SO_4_ (20%, v/v) was added to the mixture to terminate the catalytic reactions, and the absorbance of the reacted solution was recorded at 450 nm by a multi-mode microplate reader (Synergy H1, BioTek, Winooski, VT). In succession, time-dependent absorbance curves were obtained by varying the incubation time (0–20 min) in the acetate buffer (pH 3.0), and the H_2_O_2_ dose-response curves were also plotted by varying its concentration (0–100 mM).

### Preparation of WONF-modified glassy carbon electrodes

The WONF-modified GCE was prepared by the following steps. A GCE (3-mm diameter, ALS, Japan) was sequentially polished with 0.3 μm and 0.05 μm alumina slurries. It was then rinsed with DI water and ethanol in succession, sonicated in DI water several times, and subsequently rinsed again. After the polishing steps, 1 mg of WONFs was dispersed in 2 ml of DI water. Next, 5 μl of the WONFs solution was dropped onto the GCE and dried at room temperature for 3 h. Another, 1% of Nf solution was dropped onto the GCE surface and dried at room temperature for 1 h.

### Electrochemical measurements

The electrochemical measurements were conducted on a CHI 750E instrument (CH Instruments, Austin, TX) using a conventional three-electrode cell. The WONF-modified GCE was used as a working electrode. The reference electrode was Ag/AgCl, and the counter electrode was a platinum electrode. The CV and chronoamperometry measurements were conducted in 20 mM acetate buffer (pH 3.0). The CVs were recorded from −0.6 to +0.2 V at a sweep rate of 20 mV/s. The chronoamperometry measurements were recorded under an applied potential of −0.25 V (vs. Ag/AgCl).

## Additional Information

**How to cite this article**: Park, C. Y. *et al*. Hexagonal tungsten oxide nanoflowers as enzymatic mimetics and electrocatalysts. *Sci. Rep.* **7**, 40928; doi: 10.1038/srep40928 (2017).

**Publisher's note:** Springer Nature remains neutral with regard to jurisdictional claims in published maps and institutional affiliations.

## Supplementary Material

Supplementary Information

## Figures and Tables

**Figure 1:**
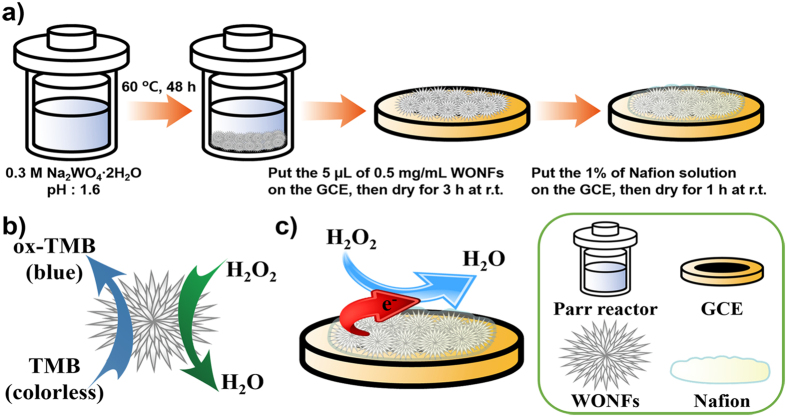
Schematic illustration in this study. (**a**) Preparation of WONFs and GCE modification. Mechanism for (**b**) the optical detection of H_2_O_2_ using TMB and (**c**) the electrocatalytic oxidation of H_2_O_2_ using the WONF-modified GCE.
