# Supplementary material for: Hexagonal tungsten oxide nanoflowers as enzymatic mimetics and electrocatalysts
Source: Sci Rep. 2017 Jan 27;7:40928. doi: 10.1038/srep40928 (PMC5269582; doi:10.1038/srep40928)
Supplement: Supplementary Information [file srep40928-s1.pdf]

Supplementary Information for

Hexagonal tungsten oxide nanoflowers as enzymatic mimetics and electrocatalysts

Chan Yeong Park,[1] Ji Min Seo,[1] Hongil Jo,[1] Juhyun Park,[2] Kang Min Ok,*[,1]

and Tae Jung Park*[,a]

[1] *Department of Chemistry, Chung-Ang University, 84 Heukseok-ro, Dongjak-gu, Seoul 06974, Republic of Korea*

[2] *School of Chemical Engineering and Materials Science, Chung–Ang University, 84 Heukseok–ro, Dongjak–gu, Seoul 06974, Republic of Korea*

* To whom correspondence should be addressed.

Email: kmok@cau.ac.kr (K.M.O.); tjpark@cau.ac.kr (T.J.P.)

1. Crystal structures of different WO$_x$

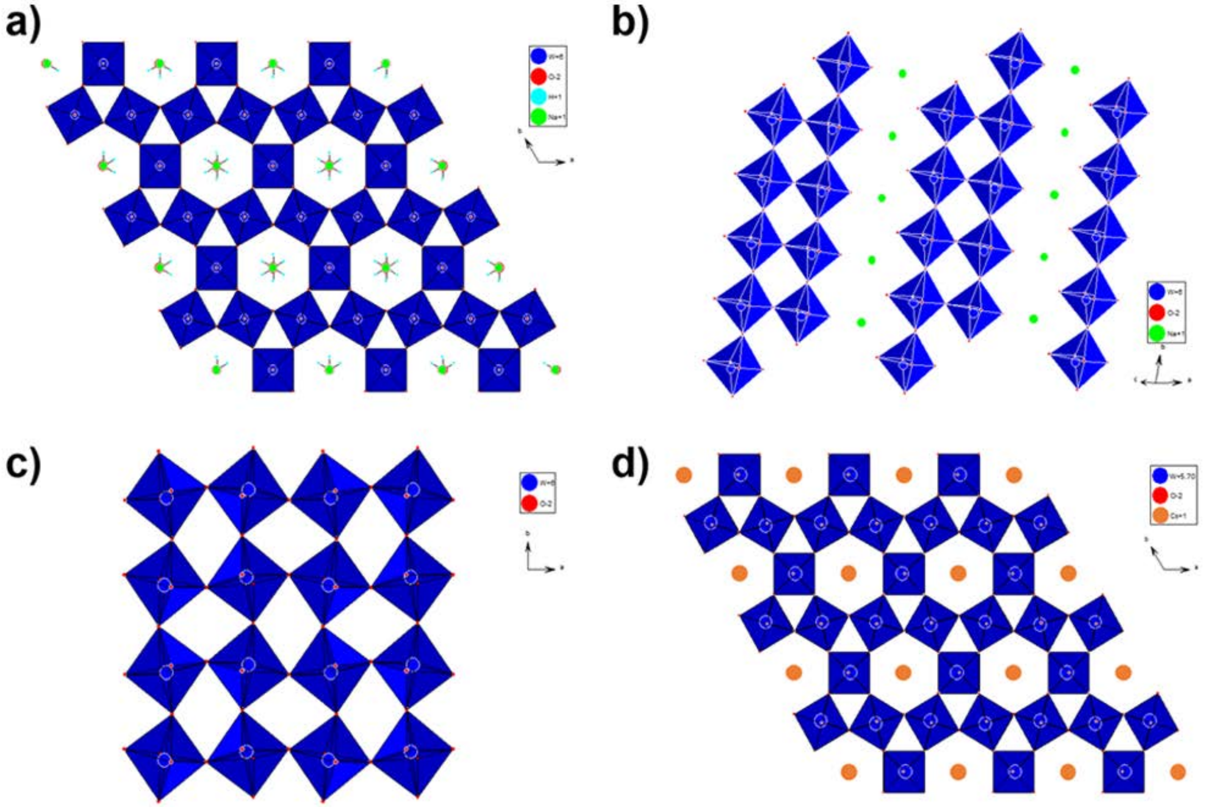

Figure S1. Crystal structure models of (a) WONFs, (b) $Na_2W_4O_{13}$ of cWONFs, (c) WO_3 of cWONFs, and (d) CsWONPs. Blue, red, green, sky blue, and orange colored spheres represent the tungsten, oxygen, sodium, hydrogen and cesium atoms.

2. TGA and DSC curves of CsWONPs

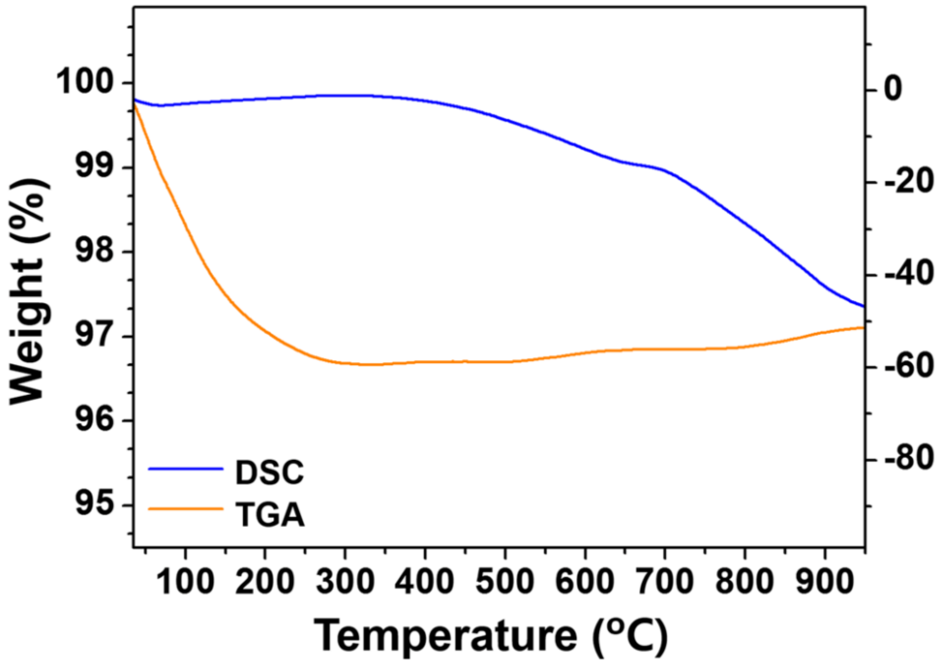

Figure S2. TGA and DSC curves of CsWONPs.

3. SEM images of different synthesized WONFs (varying the concentration of precursor)

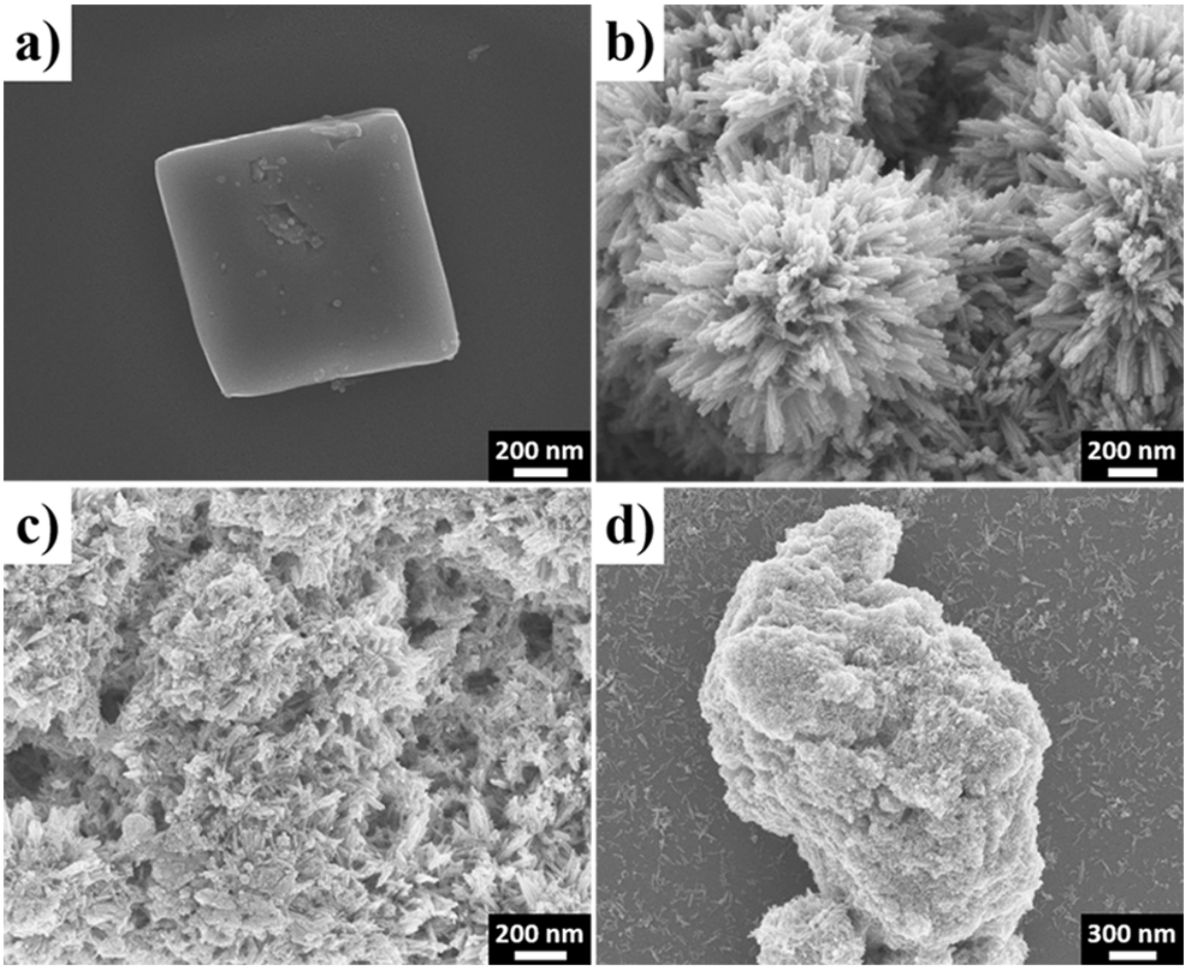

Figure S3. SEM images of different WO_x synthesized as varying the initial concentration of $Na_2WO_4{\cdot}2H_2O$. (a) 0.1 M, (b) 0.3 M, (c) 0.5 M, (d) 1.0 M.

4. XRD patterns of different synthesized WONFs (varying the concentration of precursor)

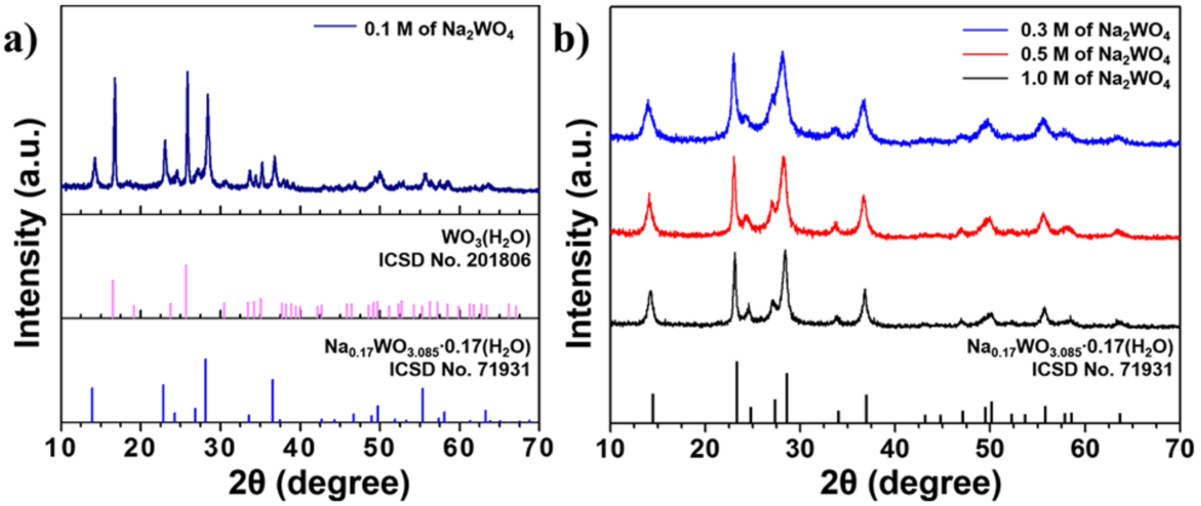

Figure S3. Powder XRD patterns of different WO_x synthesized as varying the initial concentration of $Na_2WO_4 \cdot 2H_2O$. (a) 0.1 M, (b) 0.3 M, 0.5 M, and 1.0 M.

5. SEM images of different synthesized WONFs (varying the reaction temperature)

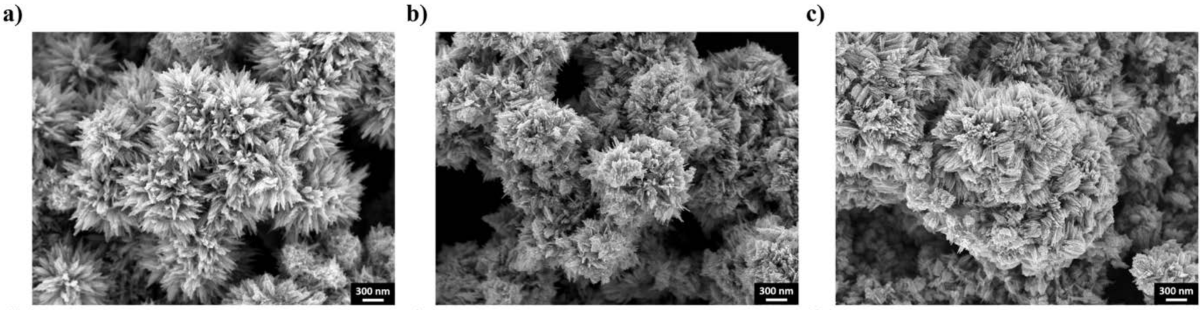

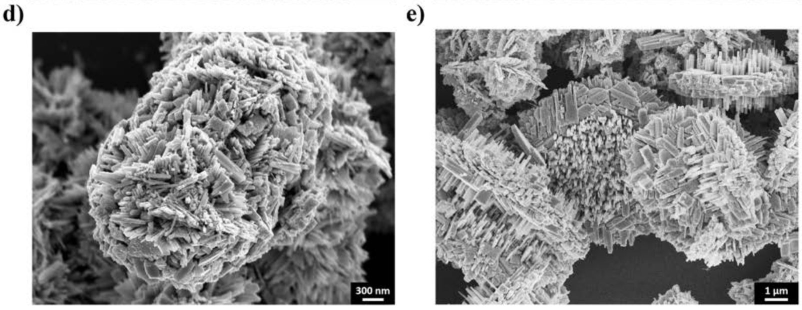

f)

Synthesis Temperature	Surface area (m^2/g)
50 °C	Not synthesized
60 °C	52
80 °C	49
100 °C	33
150 °C	8
200 °C	13

Figure S5. SEM images of different WO_x synthesized as varying the reaction temperatures. (a) 60 °C, (b) 80 °C, (c) 100 °C, (d) 150 °C, and (e) 200 °C. (f) Table for surface areas of each WO_x.

6. XRD patterns of different synthesized WONFs (varying the reaction temperature)

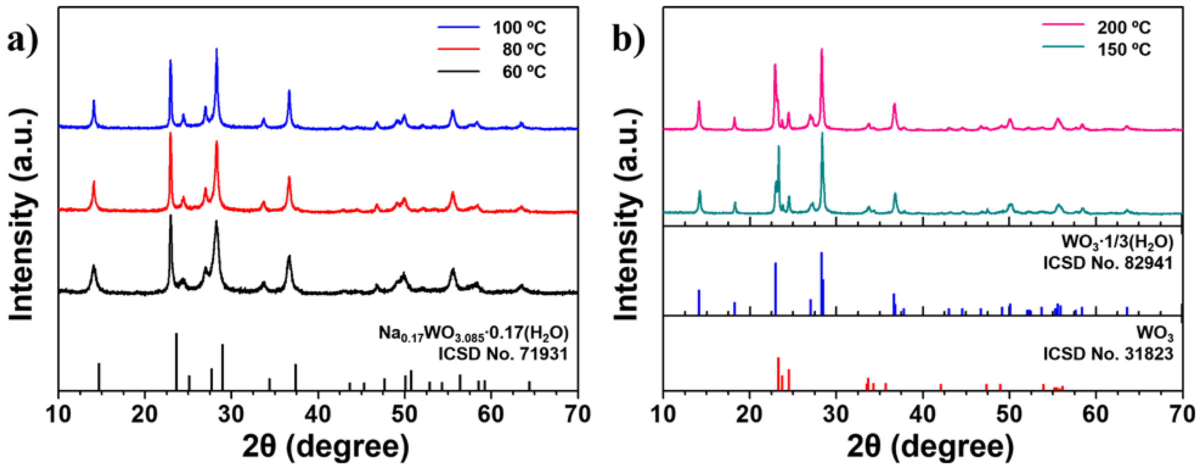

Figure S6. Powder XRD patterns of different WO_x synthesized as varying the reaction temperatures. (a) 60 °C to 100 °C, (b) 150 °C to 200 °C.

7. EDAX spectra of different WO_x

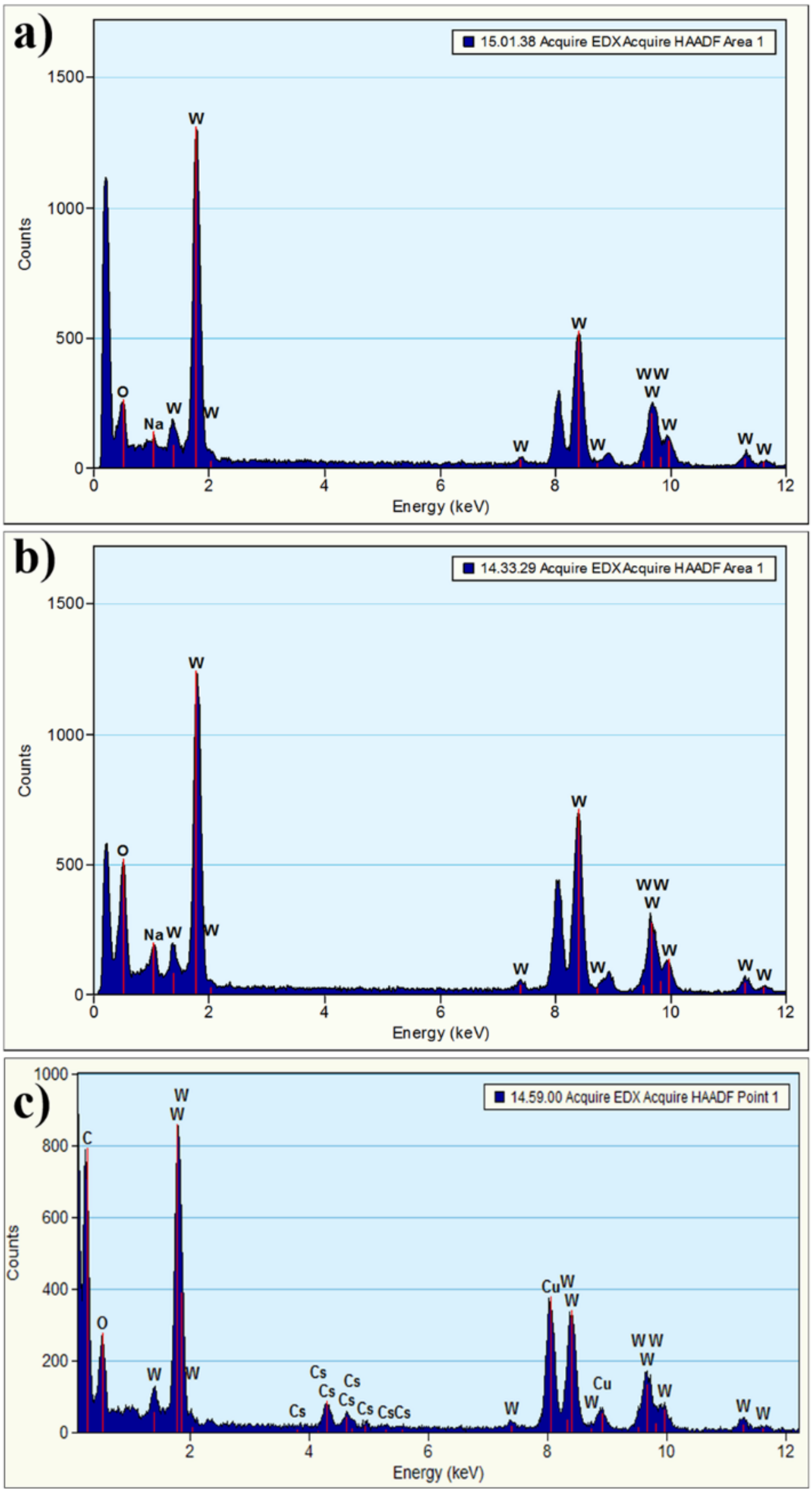

Figure S7. EDAX spectra of (a) WONFs, (b) cWONFs and (c) CsWONPs on carbon-coated copper grids.

8. XPS spectra of cWONFs

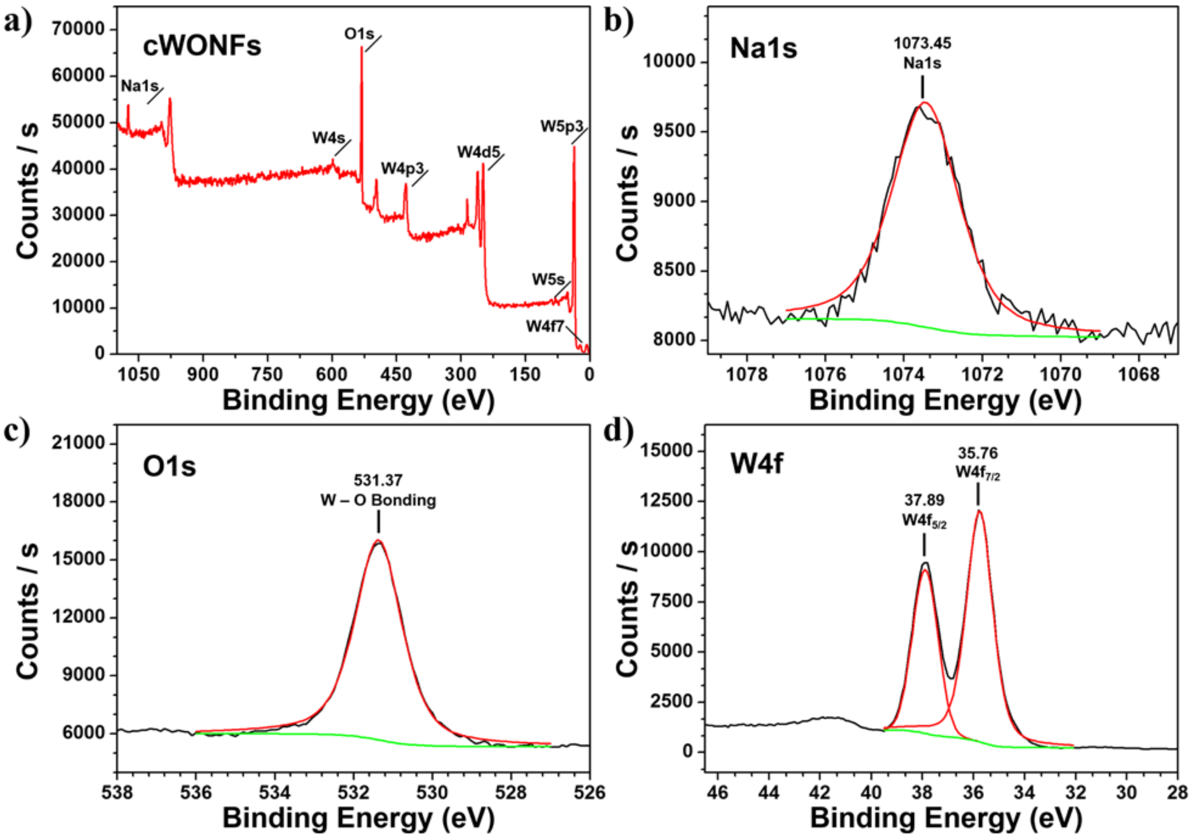

Figure S8. XPS spectra of cWONFs.

9. H_2O_2 does-response curves of cWONFs and CsWONPs

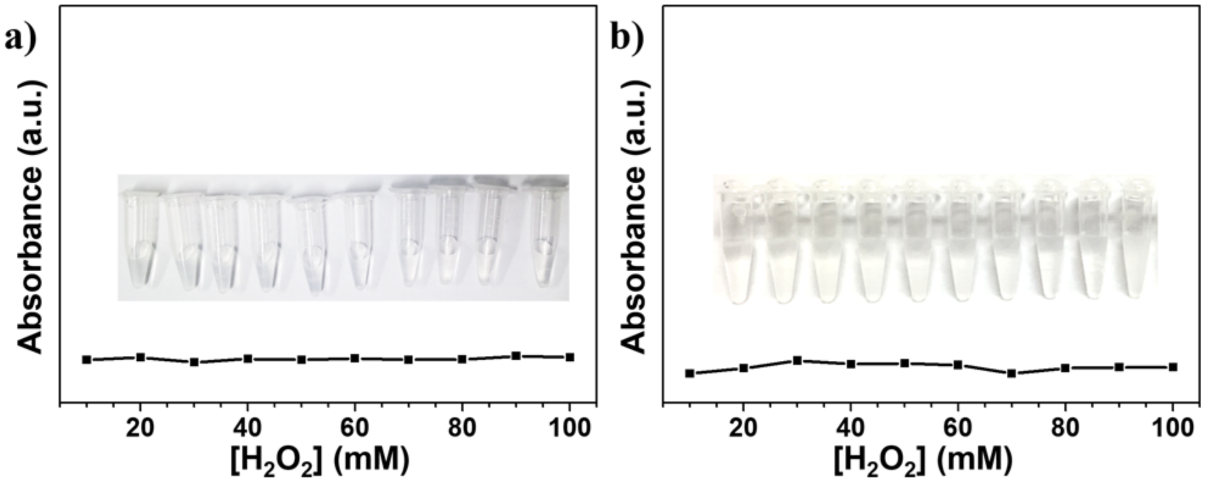

Figure S9. Dose-response curves for H_2O_2 detection using (a) cWONFs (b) CsWONPs. Inset of picture is corresponding photos for different concentrations of H_2O_2 (mM) (from left to right: 0 – 100 mM).

10. CV spectra of different modified GCE

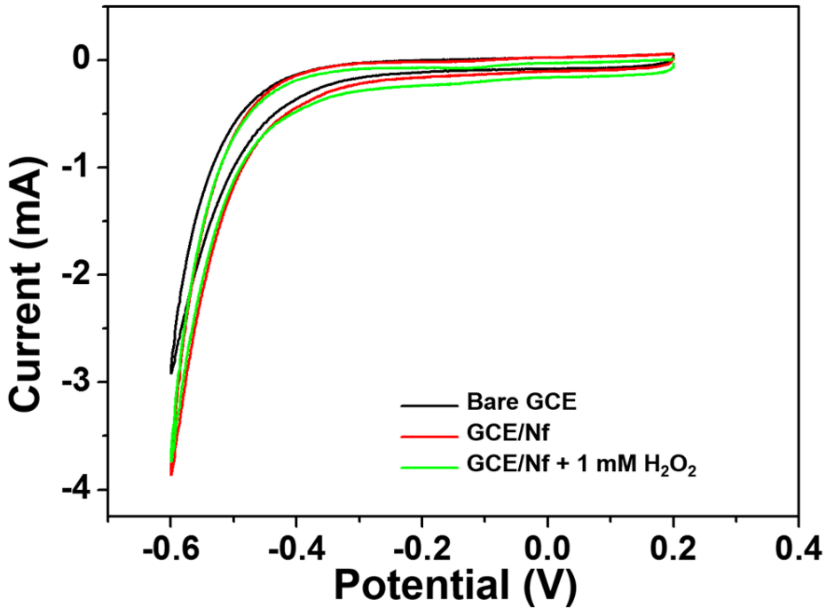

Figure S10. CV spectra of GCE, GCE/Nf and GCE/Nf + 1 mM H_2O_2.

11. Electrochemical analysis for optimal pH evaluation

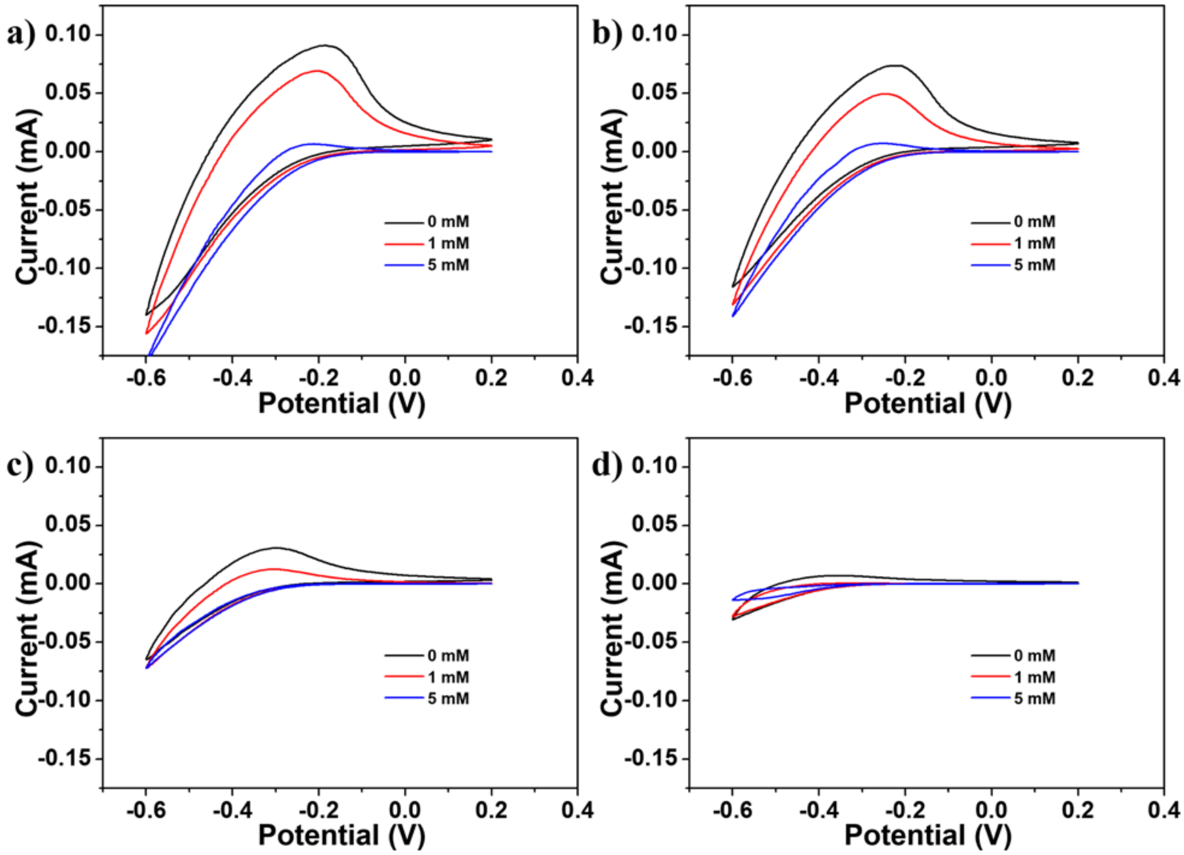

Figure S11. CV spectra of WONFs/Nf/GCE, WONFs/Nf/GCE + 1 mM H_2O_2 and WONFs/Nf/GCE + 5 mM H_2O_2 in (a) pH 3.0; (b) pH 4.0; (c) pH 5.0; (d) pH 6.0.

12. Electric reduction current plots for optimal pH verification

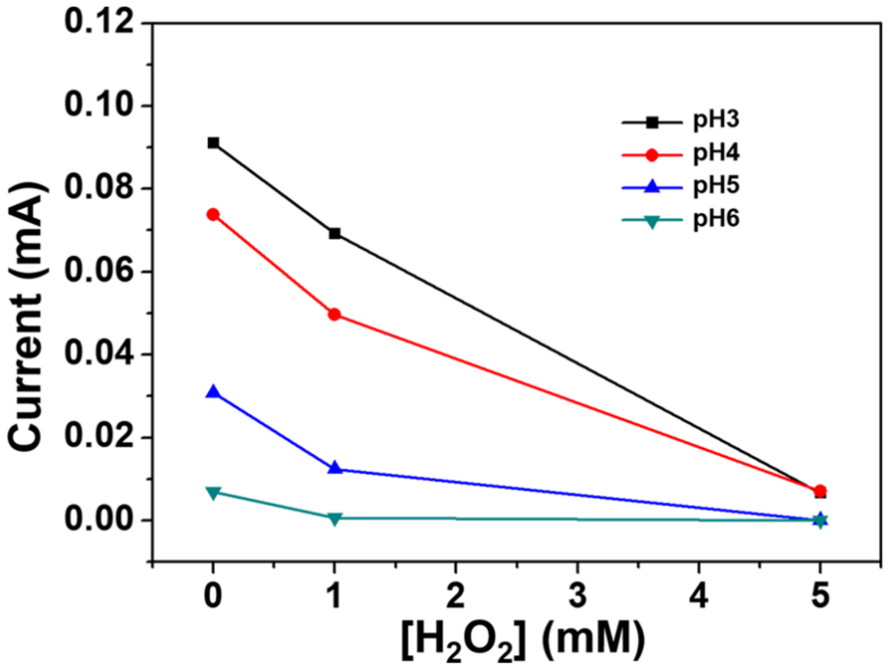

Figure S12. Reduction peak currents at different pH levels with adding H_2O_2.

13. Electrocatalytic activity of cWONFs and CsWONPs

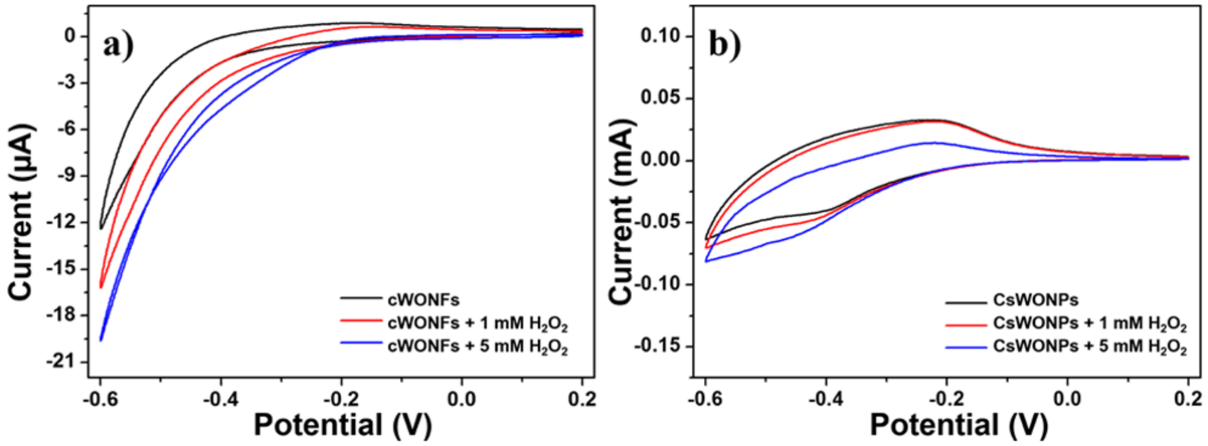

Figure S13. CV spectra of (a) cWONFs/Nf/GCE, cWONFs/Nf/GCE + 1 mM H_2O_2, cWONFs/Nf/GCE + 5 mM H_2O_2, and (b) CsWONPs/Nf/GCE, CsWONPs/Nf/GCE + 1 mM H_2O_2, CsWONPs/Nf/GCE + 5 mM H_2O_2.

Table S1. Comparison of the surface area of different shaped WONFs

Sample name	Shape	BET surface area ($m^2\ g^{-1}$)	Reference
WO_3–1.5	Flower	6	Xu et al., 2014[S1]
WO_3–2.0	Wheel	12	Xu et al., 2014[S1]
WO_3–2.5	Bundle of rods	25	Xu et al., 2014[S1]
WO_3–3.0	Rods	8	Xu et al., 2014[S1]
WONFs	Flower	52	This work
cWONFs	Agglomerated particles	3	This work
CsWONPs	Nanoparticles	33	This work

Table S2. Comparison of the kinetic parameters of the oxidation reaction catalyzed by the WONFs and previously reported $AgVO_3$, Fe_3O_4 MNPs, $ZnFe_2O_4$ MNPs, Co_3O_4 NPs, GO-COOH, and HRP, respectively

Catalyst	Substrate	k_m (mM)	v_{max} (10^{-8} M s^{-1})	Reference
HRP	H_2O_2	3.70	8.71	Wu et al., 2011[S2]
$AgVO_3$	H_2O_2	14.00	-	Xiang et al., 2016[S3]
Fe_3O_4 MNPs	H_2O_2	154.00	9.78	Gao et al., 2007[S4]
$ZnFe_2O_4$ MNPs	H_2O_2	1.66	7.74	Su et al., 2012[S5]
Co_3O_4 NPs	H_2O_2	140.07	12.10	Mu et al., 2012[S6]
GO–COOH	H_2O_2	3.99	1.24	Song et al., 2010[S7]
WONFs	H_2O_2	1.44	32.26	This work

Supplementary references

S1. Xu, D. D. *et al.* pH-dependent assembly of tungsten oxide three-dimensional architectures and their application in photocatalysis. *ACS Appl. Mater. Interfaces* **6**, 9321-9327 (2014).

S2. Wu, Y. *et al.* Ultra-small particles of iron oxide as peroxidase for immunohistochemical detection. *Nanotechnol.* **22**, 225703 (2011).

S3. Xiang, Z., Wang, Y., Ju, P. & Zhang, D. Optical determination of hydrogen peroxide by exploiting the peroxidase-like activity of $AgVO_3$ nanobelts. *Mikrochim. Acta* **183**, 457-463 (2016).

S4. Gao, L. Z. *et al.* Intrinsic peroxidase-like activity of ferromagnetic nanoparticles. *Nat. Nanotechnol.* **2**, 577-583 (2007).

S5. Su, L. *et al.* Colorimetric detection of urine glucose based $ZnFe_2O_4$ magnetic nanoparticles. *Anal. Chem.* **84**, 5753-5758 (2012).

S6. Mu, J., Wang, Y., Zhao, M. & Zhang, L. Intrinsic peroxidase-like activity and catalase-like activity of Co_3O_4 nanoparticles. *Chem. Commun.* **48**, 2540-2542 (2012).

S7. Song, Y., Qu, K., Zhao, C., Ren, J. & Qu, X. Graphene oxide: intrinsic peroxidase catalytic activity and its application to glucose detection. *Adv. Mater.* **22**, 2206-2210 (2010).
